# Closed reduction of severely angulated Rockwood and Wilkins’ type C thumb Metacarpal Base fractures in children: case series

**DOI:** 10.1186/s12891-021-04665-z

**Published:** 2021-09-12

**Authors:** Fei Qiao, Dehai Qu, Lei Cheng, Fei Jiang

**Affiliations:** 1 Department of Orthopedic Surgery, Qilu Hospital of Shandong University, Jinan, 250012 Shandong China; 2 Department of Pediatric Orthopaedic, Dalian Women and Children’s Medical Center (group), Dalian, 116012 Liaoning China

**Keywords:** Thumb metacarpus, Fractures, Percutaneous leverage, Children

## Abstract

**Background:**

Management of severely angulated Rockwood and Wilkins’ type C (RW-C) thumb metacarpal base fractures in children is challenging. We report experiences of percutaneous leverage reduction and dual antegrade crossing Kirschner (DACK) wire fixation in these fractures, aiming to assess the results using our reduction technique.

**Methods:**

From October 2011 to September 2015, A total of 17 patients with severely angulated RW-C thumb metacarpal base fractures were treated at our hospital. The injured arm, including the entire first ray, was immobilized with a thumb-spica cast for 4–6 weeks and evaluated radiologically and clinically. Percutaneous leverage reduction and DACK wire fixation were successfully performed for 17 patients. No patients were treated with open reduction. 16 patients were followed up for a mean of 32 months (range 24–41 months). The results were assessed using the modified Mayo score. The level of significance was set to be *p* < 0.05.

**Results:**

The patients included 9 girls (56.2%) and 7 boys (43.8%), with an average age of 10.8 years (range 7.5 to 14.0 years). Percutaneous leverage reduction and DACK wire fixation were successfully performed within an average total surgery time of 20 min (range 12–32 min). Bone union was achieved in all patients within a mean time of 4.2 weeks (range 4–6 weeks). The average angulation (preoperation: 50.5° (range 40.8°–67.0°) vs postoperation: 5.0° (range 0.0°–7.0°)) significantly changed from pre to post-surgery (*P* < 0.05). The clinical outcomes were evaluated by the modified Mayo score: 15 patients had an excellent outcome, and one patient had a good outcome. Cosmetic results were described as good and satisfactory by all patients. There were no refractures and no incidences of nonunion, growth arrest in the proximal epiphysis. Only one patient suffered from a superficial infection, which was resolved after the removal of the k-wires and the administration of oral antibiotics.

**Conclusion:**

Our percutaneous leverage technique with DACK wire fixation can be successfully used to treat these fractures. This technique is simple to learn and minimally invasive, and the results are satisfactory. It may be an appropriate choice for the treatment of irreducible RW-C fractures.

## Background

Thumb metacarpal fractures in children are rare, representing 1 to 5% of hand fractures, with most occurring at the base [1–3]. There are four types of thumb metacarpal base fractures in children: type A, metaphyseal fractures; type B, Salter-Harris (S-H) type II physeal fractures with lateral angulation; type C, S-H type II physeal fractures with medial angulation; and type D, S-H type III fractures (paediatric Bennett fractures) [4–6]. Closed reduction is more difficult to perform for type C (RW-C) thumb metacarpal base fractures due to the mobility of the metacarpal base and swelling [4]. Some authors have suggested that if closed reduction is performed successfully and the result is stable, short-arm spica splint or cast immobilization is possible [4, 7–9]. Otherwise, percutaneous pinning, open reduction and internal fixation are recommended for unstable and irreducible RW-C thumb metacarpal base fractures [4, 10–12]. Several techniques have been reported, including the Iselin technique and percutaneous K-wire fixation [4–6]. Percutaneous leverage reduction techniques for irreducible RW-C thumb metacarpal base fractures have not been mentioned before. The aim of this study was to assess our results treating severely angulated RW-C thumb metacarpal base fractures using the percutaneous leverage technique and dual antegrade crossing Kirschner wire (DACK wire) fixation.

## Methods

### Patients

This study was approved by the Institutional Ethical Review Board of Dalian Women and Children’s Medical Center (group) (approval number 20003). Written informed consent was obtained from all guardians for anonymized data analysis and publication. A total of 17 patients with severely angulated RW-C thumb metacarpal base fractures were treated at our hospital from October 2011 to September 2015. A total of 16 patients were followed up for a mean of 32 months (range 24–41 months). All cases were classified as severely angulated RW-C fractures. All surgeries were performed by the senior surgeon, and the average surgery time was 20 min (range 12–32 min). The injured arm, including the entire first ray, was immobilized with a thumb-spica cast for 4–6 weeks and evaluated radiologically and clinically.

### Surgical procedures

**Fig. 1:**
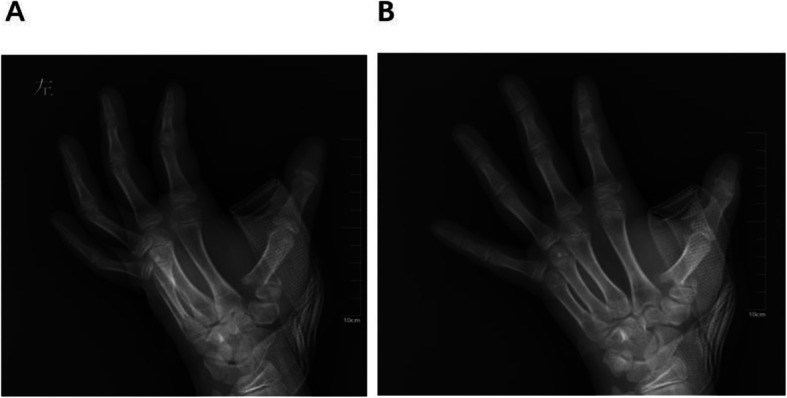
Typical thumb metacarpal base RW-C fracture of left hand. **a** AP X-Ray of left thumb preoperative. **b** lateral X-Ray of left thumb preoperative

**Fig. 2:**
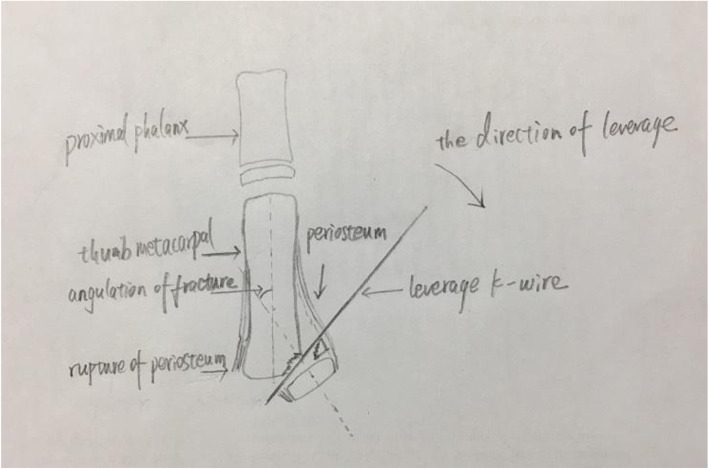
Diagram of percutaneous leverage of RW-C fracture of left hand (drawn by Dr. Qiao)

**Fig. 3:**
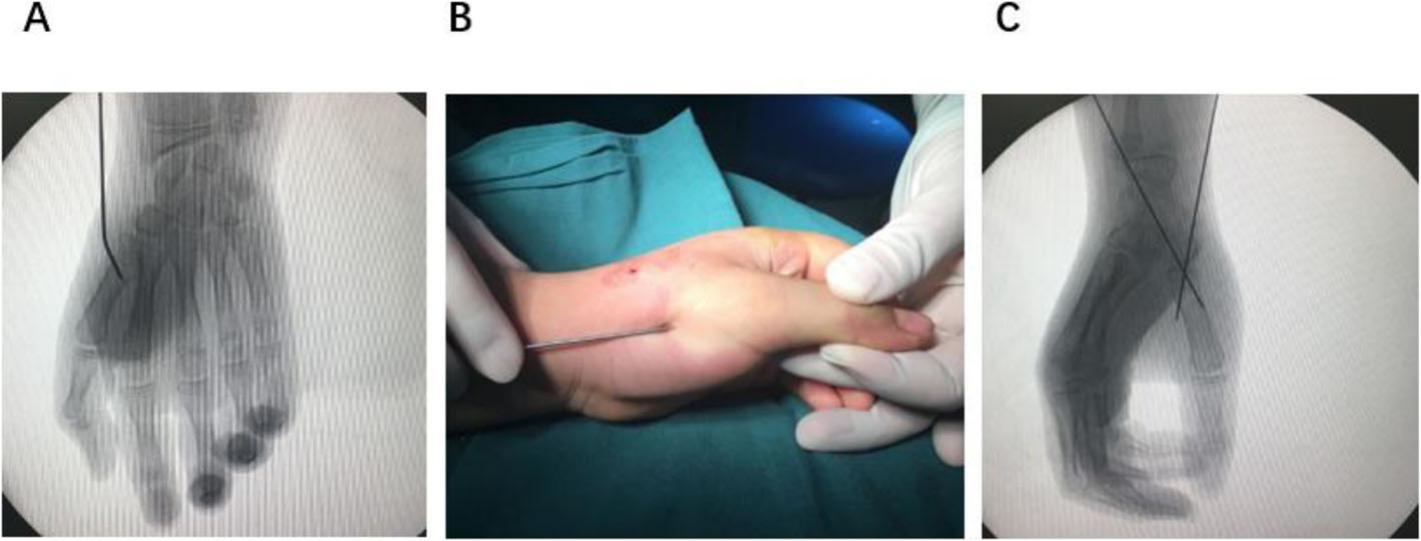
Typical thumb metacarpal base RW-C fracture of left hand. **a** leverage reduction of the fracture. **b** aspect of leverage reduction. **c** C-arm result after pinning

After induction of general anaesthesia, with a C-arm image intensifier, a leverage K-wire with a 1.5 mm diameter was radialis percutaneously inserted into the bone fragment (Figs. 1, 2). The procedure was performed carefully so that the wire did not penetrate too deeply past the dorsal cortex of the distal fragment. Once the K-wire crossed the fracture site, it was moved into position so that its tip could be moved towards the metaphysis to prevent injury to the physis, and supplementary pressure was placed on the dorsal and medial rims of the distal fragment for reduction. After the fracture reduction was confirmed, especially as seen on the anteroposterior and lateral radiographs (Fig. 3a, b). Anatomic reduction was maintained with DACK wires measuring 1.0 mm in diameter (Fig. 3c). After successful reduction and fixation, the external part of the wire was bent to an angle of 90°. The injured arm, including the entire first ray, was immobilized with a thumb-spica cast for 4–6 weeks; when the wires and cast were removed at the outpatient department, continuous passive motion (CPM) was encouraged.

### Postoperative evaluation

**Fig. 4:**
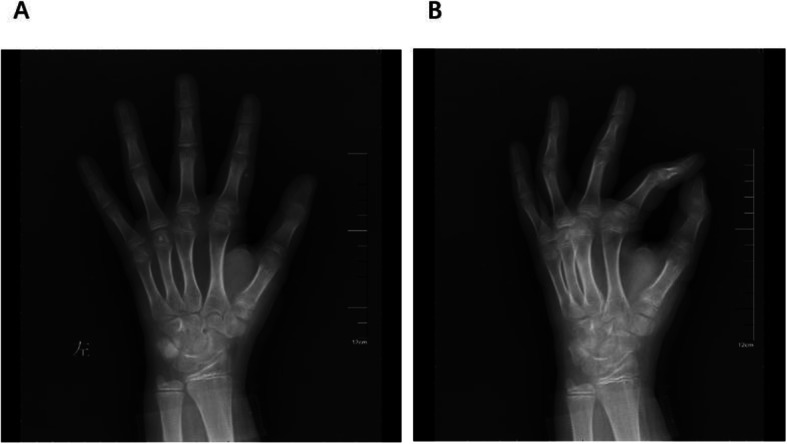
Typical thumb metacarpal base RW-C fracture of left hand.8 weeks follow-up X ray

The first clinical review was conducted 2 weeks after surgery. Then, the patients were assessed radiographically for fixation and bone union 4 weeks, 6 weeks, 8 weeks and 6 months postoperatively and every 6 months thereafter. When calluses formed, the cast and K-wires were removed without anaesthesia, and active exercise was encouraged to recover the full range of motion (ROM) of the thumb. The average follow-up duration was 32 months (range 24–41 months). The results were assessed using the modified Mayo score [13] (Table 1) (Fig. 4).

**Table 1:** Modified Mayo Score^a^

Category	Points	Examination Findings
Pain	25	No pain
	20	Pain only with weather change
	15	Moderate pain on exertion
	15	Slight pain with activities of daily living
	5	Moderate pain with activities of daily living
	0	Pain at rest
Satisfaction	25	Very satisfied
	20	Moderately satisfied
	10	Unsatisfied but fit for work
	0	Unsatisfied and unfit for work
ROM ^**b**^(IP, MCP, saddle joint)	25	100% of the uninjured thumb
	15	75–99% of the uninjured thumb
	10	50–74% of the uninjured thumb
	5	25–49% of the uninjured thumb
	0	0–24% of the uninjured thumb
Pulp-to-palm distance ^**c**^	25	100% of the uninjured thumb
	15	75–99% of the uninjured thumb
	10	50–74% of the uninjured thumb
	5	25–49% of the uninjured thumb
	0	0–24% of the uninjured thumb
Power measurement ^**d**^	25	100% of the uninjured thumb
	15	75–99% of the uninjured thumb
	10	50–74% of the uninjured thumb
	5	25–49% of the uninjured thumb
	0	0–24% of the uninjured thumb
Sensibility^**e**^	25	Normal sensibility
	20	Diminished light touch
	15	Diminished protective sensation
	10	Loss of protective sensation
	5	Deep sensation of pressure
	0	Without sensation
Final score (points)	135–150	Excellent
	120–134	Good
	97–119	Fair
	< 97	Poor

^a^Reference: Parvizi D, Haas FM. Division of Plastic, Aesthetic and Reconstructive Surgery, Department of Surgery, Medical University Hospital of Graz, Austria

^b^Rang of movement (sum of IP, MCP, and saddle joint)

^c^Defined as the distance of the thumb pulp to the metacarpophalangeal furrow of the fifth digit in centimeter

^d^Sum of adduction and pinch grip

^e^By Semmes-Weinstein monofilaments

A score of 97 points or better was considered to be a “satisfactory result”

### Statistical analysis

SPSS v22 (IBM Corp., Armonk, NY, USA) was used for statistical analysis. For nonnormally distributed data, the Mann-Whitney U test for independent samples was conducted. For normally distributed data, the paired-samples *t* test was used to assess differences between the preoperative and postoperative results. The level of significance was set to *p* < 0.05.

## Results

The patients included 9 girls (56.2%) and 7 boys (43.8%), with an average age of 10.8 years (range 7.5 to 14.0 years). Female patients (mean age 10.3 years, range 8.0 to 12.0 years) were younger than male patients (mean age 11.5 years, range 7.5 to 14.0 years; *p* = 0.957, Mann-Whitney U test). The most common causes of these fractures were falls and low-impact sports (81.3%, *n* = 13/16). In all, 37.5% (*n* = 6/16) of the fractures were left-sided injuries, and 62.5% (*n* = 10/16) were right-sided injuries. None of the injuries was bilateral. All were S-H II lesions, and none were open fractures. Percutaneous leverage reduction and DACK wire fixation were successfully performed within an average total surgery time of 20 min (range 12–32 min). Bone union was achieved in all patients within a mean time of 4.2 weeks (range 4–6 weeks). The average angulation (preoperation: 50.5° (range 40.8°–67.0°) vs postoperation: 5.0° (range 0.0°–7.0°)) significantly changed from pre to post-surgery (*P* < 0.05). Only one patient experienced a superficial infection, which was resolved after the removal of the K-wires and the administration of oral antibiotics. The clinical outcomes were evaluated by the modified Mayo score: 15 patients had an excellent outcome, and one patient had a good outcome. There were no cases of deep infection, secondary displacement, malunion, or growth arrest in the proximal physis (after at least 2 years follow-up). No nerve injury or post-traumatic osteoarthritis occurred during the follow-up period. All 16 patients recovered full mobility of the first ray with respect to that on the contralateral side (Table 2).

**Table 2:** The details of the patients and fractures

Demographic	Total(***n*** = 16)	***p***-value
Sex, n (%)		
Female	9 (56.2%)	
Male	7 (43.8%)	
Mean age (yrs)		0.957^a^
Female	10.3	
Male	11.5	
Trauma mechanism, n (%)		
Fall/low-energy	8 (50.0%)	
Sport activity	5 (31.25%)	
Fall> 2 m	1 (6.25%)	
Traffic accident	2 (12.5%)	
Polytrauma	0	
Fracture classification		
Salter-Harris I	0	
Salter-Harris II	16	
Close fractures	16	
Open fractures	0	
Mean BMI kg/m^2^		0.633^a^
Female	22.3	
Male	23.0	
Mean Angulation(°)		0.000^b^
Preoperation	50.5°	
Postoperation	5.0°	

^a^Mann-Whitney U test

^b^Paired-samples *t* test

Statistically significance was set to be *p* < 0.05

## Discussion

Obviously displaced RW-C thumb metacarpal base fractures are rare in children, and their management remains challenging for paediatric orthopaedic clinicians. This is the first observational study of these fractures in children treated with percutaneous leverage reduction and DACK wire fixation. Our study shows that the described method of fixation for these fractures achieves satisfactory results. All children achieved radiographic union at a mean time of 4.2 weeks and pain-free complete movement after K-wire removal. In our series of 16 patients, no open operative reduction was performed. Secondary displacement of the reduction was not observed.

Based on the potential for remodelling growing bones, the indication for aggressive treatment remains controversial. According to some authors, RW-C fractures with less than 30 degrees of angulation disbalance can be treated by closed reduction and splinting. Ruptures of the dorsal and medial periosteum make the fracture unstable, and thumb-spica cast immobilization with the first ray yields unreliable results [6]. Some researchers have recommended that aggressive procedures be performed in children when the maximum angle of the fracture is > 30 degrees, the magnitude of displacement of the fracture is > 2/3 of the diameter of the growth plate, or a rotational deformity is present [6, 8, 9]. In fact, the mean angulations in our research indicating the need for aggressive procedures were considerably higher than the given limits (average 50.5°, range 40.8°–67.0°). Thus, the indications of acceptability for imperfect reductions exist for two reasons. First, the first ray comprises a series of joints that can compensate for small extra-articular displacements without causing severe disability. Second, such displacements can be corrected by remodelling the growth plate [14, 15]. However, this spontaneous correction requires 2 years [5]. We must remember that the growth plate closes at an average age of 14.5 years in girls and 16.5 years in boys when considering the indications for the treatment of these fractures [6, 16].

In this study, our technique was demonstrated to be a minimally invasive, rapid, and technically uncomplicated method for treating these fractures. Manual closed reduction of RW-C fractures requires axial traction on the thumb and the placement of pressure on the base of the distal fragment [17]. The mobility of the metacarpal base and swelling make closed reduction difficult. Comminution, soft tissue interposition, or transperiosteal “buttonholing” may further complicate reduction [4, 10]. Peter M et al. indicated that when closed reduction is performed unsuccessfully, open reduction is also required [4]. Other studies have reported that severely displaced RW-C fractures might require open reduction to remove any portions of interposed periosteum that could prevent reduction. Open reduction is indicated for irreducible RW-C fractures [4, 12]. In our study, no open reduction was needed, and excellent and good clinical outcomes, as evaluated by the modified Mayo score, were achieved for 15 and 1 patient, respectively.

The number of leverage-based manual reduction attempts can be reduced to fewer than 3, while injury to the physis caused by the tip of the leverage K-wire can be avoided. In general, manual reduction and leverage treatment for paediatric fractures, including S-H type II fractures of the distal radius, radial neck fractures, supracondylar fractures, and Bennett fractures, are successful and yield good results [18–21]. We first conducted leverage reduction to anatomically reduce the fractures in our study. For at least 2 years of follow-up, there were no cases of injury to the extensor tendons, secondary displacement, premature physis closure, bone bridge formation or epiphyseal ischaemic necrosis in our study.

There are many pin configuration options, including pinning across the reduced carpometacarpal (CMC) joint, the Iselin technique, the modified Iselin technique, and direct fixation across the fracture [6, 12, 22–24]. Some authors have shown that intraarticular K-wires may aggravate articular surface lesions and cause posttraumatic arthritis. Thus, the Iselin method was proposed [25]. Some researchers have determined the incidence of secondary displacement resulting from a faulty Iselin technical approach and a decrease in the quality of reduction [17]. Wiggins preferred transfixing a K-wire across the epiphyseal growth plate, which has never been reported to cause epiphysiodesis [26]. Hastings also demonstrated that thumb base fracture fixation with longitudinal K-wire fixation yields good results [22]. We prefer DACK wire fixation, whose advantages include the easy selection of the needle puncturing point and the stable transfixion of K-wires across the epiphyseal growth plate, which yields higher stability than the Iselin technique.

In summary, our clinical results for DACK wire fixation compare favourably with those of previous studies. Jehanno et al. [6] reported on a non-operative treatment for RW-C fractures, in which early secondary displacement occurred in 2 out of 4 patients. Niekerk et al. [27] reported complications in 2 of 4 patients treated by the Iselin technique, including one slight complication and one severe complication of interference with daily activities, hobbies and sports. Greeven A et al. [28] reported on a series of 15 patients with extra-articular fractures of the thumb metacarpal base treated by the Iselin technique; in three patients, pin tract infections occurred, requiring treatment with oral antibiotics. Two other patients suffered severe complications such as an inability to carry out a previous hobby and a loss of grip strength compared with the contralateral side. We did not experience any severe complications, except one pin tract infection, in our series of 16 patients.

In our research, most of the leverage procedures were performed within 0.30 min with 1–3 leverage attempts. A longer-duration leverage procedure is associated with more radiation exposure (RE). The risk of RE needs to be understood and minimized in paediatric trauma theatres, as RE is associated with malignant diseases [29]. Ultrasonography (US) has also been used for intraoperative monitoring for the treatment of radial neck fractures in children to reduce the dose of RE [30]. US could be a useful alternative to X-ray in the future for this kind of fracture during intraoperative intensification.

Our results show that the following key points should be understood when performing the procedures: (1) According to the preoperative imaging and C-arm image intensifier data, the plane with the largest fracture displacement and angulation should be chosen as the leverage plane to achieve anatomical reduction and reduce the number of leverage attempts. (2) The abductor pollicis longus tendon and the first metacarpal epiphysis should be considered for the wire puncturing points to reduce tendinous adhesion. (3) DACK wire fixation is more reliable than other forms of fixation. (4) When leveraging, the tip of the K-wire should be moved towards the metaphysis to prevent injury to the physis. (5) In contrast to the other metacarpals, the thumb metacarpal is visible on both AP and lateral X-rays, and the angulation and displacement can be assessed more reliably.

The main limitation of this study is that it is a retrospective cohort study with a small sample size. Additional studies with larger sample sizes, longer follow-up periods, and control groups are needed. It would be also valuable to compare our technique with open reduction.

## Conclusions

Our percutaneous leverage technique with DACK wire fixation can be successfully used to treat these fractures. This technique is simple to learn and minimally invasive, and the results are satisfactory. It may be an appropriate choice for the treatment of irreducible RW-C fractures.

## Data Availability

All data generated and/or analyzed during the current study are available in this published article. Data required that are not in the article are available from the corresponding author on reasonable request.

## Abbreviations

RW-C: Rockwood and Wilkins’ type C
DACK wire: Dual antegrade crossing Kirschner wire
S-H: Salter-Harris
CPM: Continuous passive motion
CMC: Carpometacarpal
3DCT: Three-dimensional computerized tomography
US: Ultrasonography
RE: Radiation exposure
ROM: Rang of movement
IP: Interphalangeal
MCP: Metacarpophalangeal
BMI: Body Mass Index
